# Oestradiol synthesized by female neurons generates sex differences in neuritogenesis

**DOI:** 10.1038/srep31891

**Published:** 2016-08-24

**Authors:** Isabel Ruiz-Palmero, Ana Ortiz-Rodriguez, Roberto Cosimo Melcangi, Donatella Caruso, Luis M. Garcia-Segura, Gabriele M. Rune, Maria-Angeles Arevalo

**Affiliations:** 1 Instituto Cajal, Consejo Superior de Investigaciones Científicas (CSIC), Avenida Doctor Arce 37, 28002 Madrid, Spain; 2 Dipartimento di Scienze Farmacologiche e Biomolecolari, Center of Excellence on Neurodegenerative Diseases, Università degli Studi di Milano, Via G. Balzaretti 9, 20133 Milan, Italy; 3 Institute of Neuroanatomy, University Medical Center Hamburg-Eppendorf, Martinistr. 52, 20246 Hamburg, Germany

## Abstract

Testosterone produced by the foetal testis is converted by male neurons to oestradiol, which masculinizes neuronal morphology. Female neurons are known to synthesize oestradiol in absence of exogenous testosterone. However, the role of neuronal oestradiol on the differentiation of foetal female neurons is unknown. Here we show that, due to endogenous neuronal oestradiol synthesis, female hippocampal neurons have higher expression of the neuritogenic protein Neurogenin 3 and enhanced neuritogenesis than males. Exogenous application of testosterone or its metabolite dihydrotestosterone increases Neurogenin 3 expression and promotes neuritogenesis in males, but reduces these parameters in females. Together our data indicate that gonadal-independent oestradiol synthesis by female neurons participates in the generation of sex differences in hippocampal neuronal development.

The brain is a target for sex steroid hormones, such as testosterone and oestradiol, which are synthesized in the gonads and transported to the brain via circulation. In the male brain, testosterone produced by the foetal testis is converted in oestradiol, which regulates several aspects of neuronal development, including neuritogenesis^1^,^2^, essential for the adequate generation of functional neuronal circuits. In addition, the brain is a steroidogenic tissue and expresses the steroidogenic molecules and enzymes necessary for the formation of testosterone from cholesterol^3^. In turn testosterone is metabolized within the brain in oestradiol by aromatase and in dihydrotestosterone (DHT) by 5α-reductase^3^.

Brain sex differentiation in rodents depends on a peak in testosterone production by the foetal testis at embryonic days 17–18^4^,^5^. The masculinising effects of testosterone in the brain are mediated by its metabolites oestradiol and DHT^2^,^6^. While the role of testicular testosterone in brain sex differentiation has been extensively studied, little is known on the role of endogenous brain steroid synthesis in this process. Previous studies have shown that the development of hypothalamic and mesencephalic neurons in primary cultures is sexually dimorphic^7^,^8^,^9^,^10^,^11^. In hypothalamic cultures, prepared before the peak of testosterone production by the foetal testis, female neurons develop faster than male neurons and this is associated with a higher expression of the neuritogenic factor Neurogenin 3 (Ngn3; Neurog3) in female neurons^7^. In this study, we have explored the expression of Ngn3 and neuritogenesis in primary mouse hippocampal cultures of male and female embryos in order to determine potential sex differences resulting from the effects of endogenous steroid synthesis.

## Results

### Sex differences in neuritogenesis

**Figure 1:**
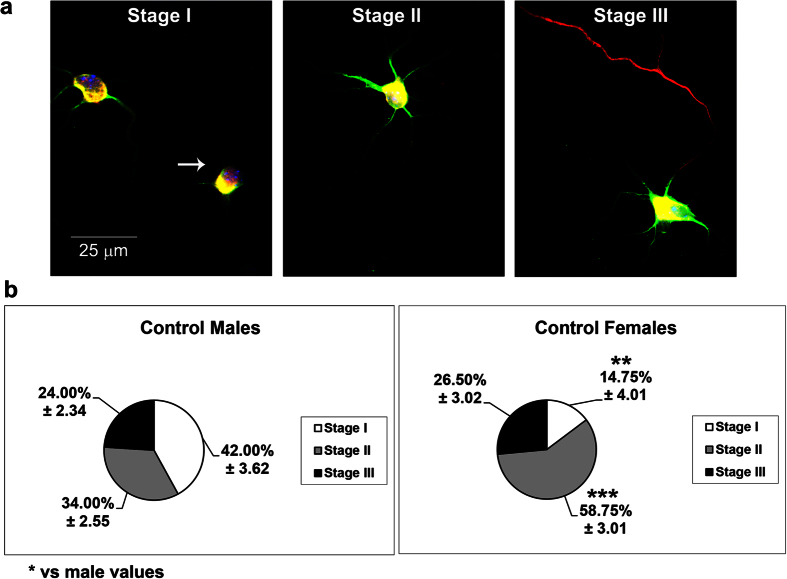
Proportion of hippocampal neurons in different stages of differentiation in male and female cultures at 2 days *in vitro* (DIV). (**a**) Representative examples of the three stages of neuronal differentiation observed in male and female hippocampal cultures at 2 DIV. Stage I corresponds to a neuron without neurites and axon (indicated by the arrow); Stage II corresponds to a neuron with primary neurites or defined dendrites, but without axon; Stage III corresponds to a neuron with high or low number of dendrites and a defined axon. Neurons are immunostained for microtubule associated protein-2 (MAP2) (green), TAU (red) and DAPI (blue). (**b**) Proportion of neurons in the three stages of differentiation in male and female cultures, under basal conditions. Data are the mean ± SEM of 4 hippocampal cultures per sex. ***p < 0.001 and **p < 0.01 vs male values at the same stage.

**Figure 2:**
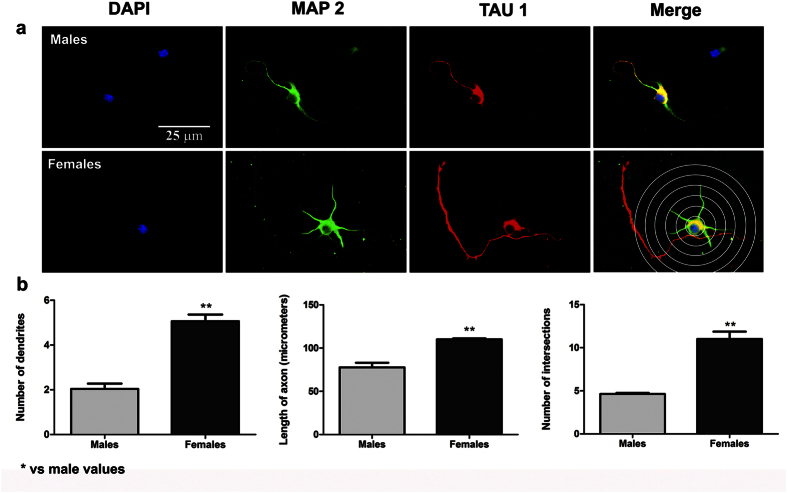
Morphological analysis of neurites in hippocampal neurons from male and female cultures under basal conditions. (**a**) Representative examples of male and female neurons on Stage III at 2 DIV. In the right bottom panel, the grid used for Sholl analysis is shown. Neurons are immunostained for microtubule associated protein-2 (MAP2) (green), TAU (red) and DAPI (blue). (**b**) Number of primary neurites, length of the axon and complexity of the dendritic arbour (number of intersections of dendrites with the circles in the Sholl analysis). Data are the mean ± SEM of 3 hippocampal cultures per sex. **p < 0.01 vs male values.

Based upon previous studies by Scerbo *et al*.^7^, who demonstrated sex-dependent neuritogenesis in primary hypothalamic neurons, we explored, as a first step, whether this is also the case in primary hippocampal neurons. After two days *in vitro* (DIV), hippocampal neurons displayed a sex-dependent degree of differentiation (Fig. 1). In cultures from males, 42.00 ± 3.62% of the cells were devoid of any neuronal process (Stage I) and 34.00 ± 2.55% of the cells showed neurites, but not a differentiated axon (Stage II). Only 14.75 ± 4.01% of the cells in female cultures were in Stage I (Student’s t-test, p = 0.002 vs male values), while 58.75 ± 3.01% of the cells were already in Stage II (p < 0.001 vs male values). The proportion of the cells showing dendrites and axons was similar in male and female cultures (24.00 ± 2.34% and 26.50 ± 2.02%, respectively; Stage III) (Fig. 1b). The morphometric analysis, considering neurons on Stages II and III, revealed that female neurons had more primary dendrites (Student t-Test, p = 0.001), longer axons (p = 0.004) and a higher dendritic complexity assessed by Sholl analysis (dendritic intersections; p = 0.002) than male neurons (Fig. 2).

### Sex differences in neuritogenesis are associated with sex differences in Ngn3 expression

**Figure 3:**
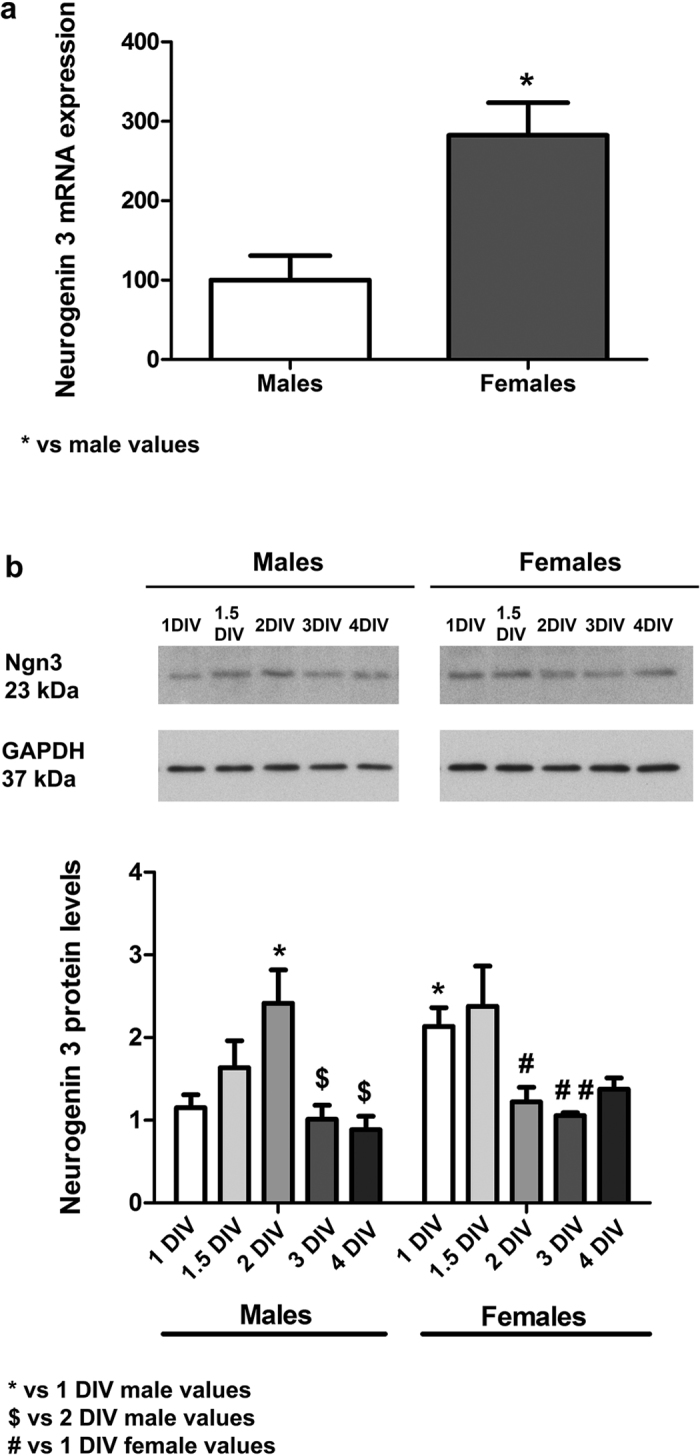
Sex differences in Neurogenin 3 (Ngn3) expression in hippocampal primary neuronal cultures. (**a**) Ngn3 mRNA levels in male and female neurons at 1 DIV. Data are the mean ± SEM of 6 hippocampal cultures. **p < 0.01 vs male values. (**b)** Ngn3 protein levels in cultures at 1, 1.5, 2, 3 and 4 DIV. Data are the mean ± SEM of 5 cultures. **p < 0.01 and *p < 0.05 vs 1 DIV male values. ^$^p < 0.05 vs 2 DIV male values. ^#^p < 0.05 vs 1 DIV female values.

**Figure 4:**
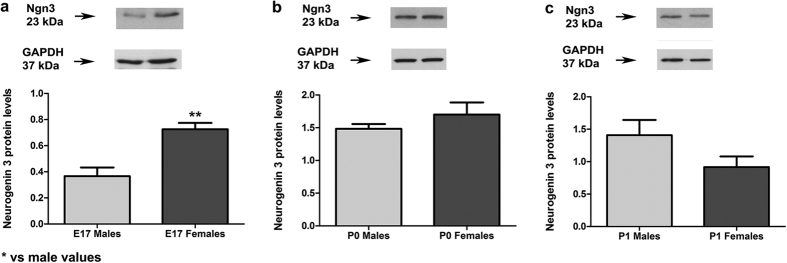
Sex differences in Neurogenin 3 (Ngn3) mouse hippocampal expression at E17, P0 and P1. (**a**) Ngn3 protein levels in E17 male and female hippocampi. Data are the mean ± SEM of 4 hippocampi per sex. **p < 0.01 vs E17 male value. (**b,c**) Ngn3 protein levels in P0 and P1 male and female hippocampi. Data are the mean ± SEM of 4 hippocampi per sex. No statistical differences in Ngn3 protein levels were found between male and female P0 or P1 hippocampi.

Previous studies in unsexed cultures have shown that Ngn3 is a neuritogenic factor in hippocampal neurons and that Ngn3 expression increases during neuronal differentiation *in vitro* and reaches a maximum during the early neuritogenic period^12^. Hence, we speculated that basal sex differences in neuritogenesis in male and female hippocampal neurons should be associated with sex differences in Ngn3 expression. As shown in Fig. 3a, basal Ngn3 mRNA levels were higher in female than in male neurons at 1 DIV (Student’s t-test, p = 0.021). A similar sex difference was also observed in the hippocampus at embryonic day 17 *in vivo* at the protein level (p = 0.005; Fig. 4a).

We examined Ngn3 protein levels in neurons of various stages of differentiation in culture (Fig. 3b). Two-way ANOVA revealed a significant effect of DIV [F(4,38) = 5.324; p = 0.002] and a significant sex*DIV interaction [F(4,38) = 4.137; p = 0.007]. The Bonferroni post hoc test revealed a significant decrease in Ngn3 protein levels in male neurons at 3 DIV (p = 0.043) and 4 DIV (p = 0.045) respect 2 DIV and a significant decrease in Ngn3 protein levels in female neurons at 2 DIV (p = 0.013) and 3 DIV (p = 0.003) respect 1 DIV. Therefore, Ngn3 protein levels initially increase progressively, reach a maximum and decrease thereafter in both male and female neurons. In female neurons, however, the expression is higher than in males at 1 DIV. The existence of a transient sex difference in Ngn3 expression was also confirmed *in vivo* (Fig. 4a–c). Higher expression of Ngn3 was detected in the hippocampus of females at embryonic day 17, compared to the hippocampus of males (Student’s t-Test, p = 0.005). This sex difference disappeared by postnatal day 0. These temporal differences in the expression of Ngn3 in the hippocampus *in vivo* may be the cause of the observed sex differences in neuronal development *in vitro*. Indeed, when cultures were prepared from hippocampus at embryonic day 18 sex differences in neuronal development were not observed (data not shown).

### Ngn3 silencing decreases neuritogenesis in male and female neurons

**Figure 5:**
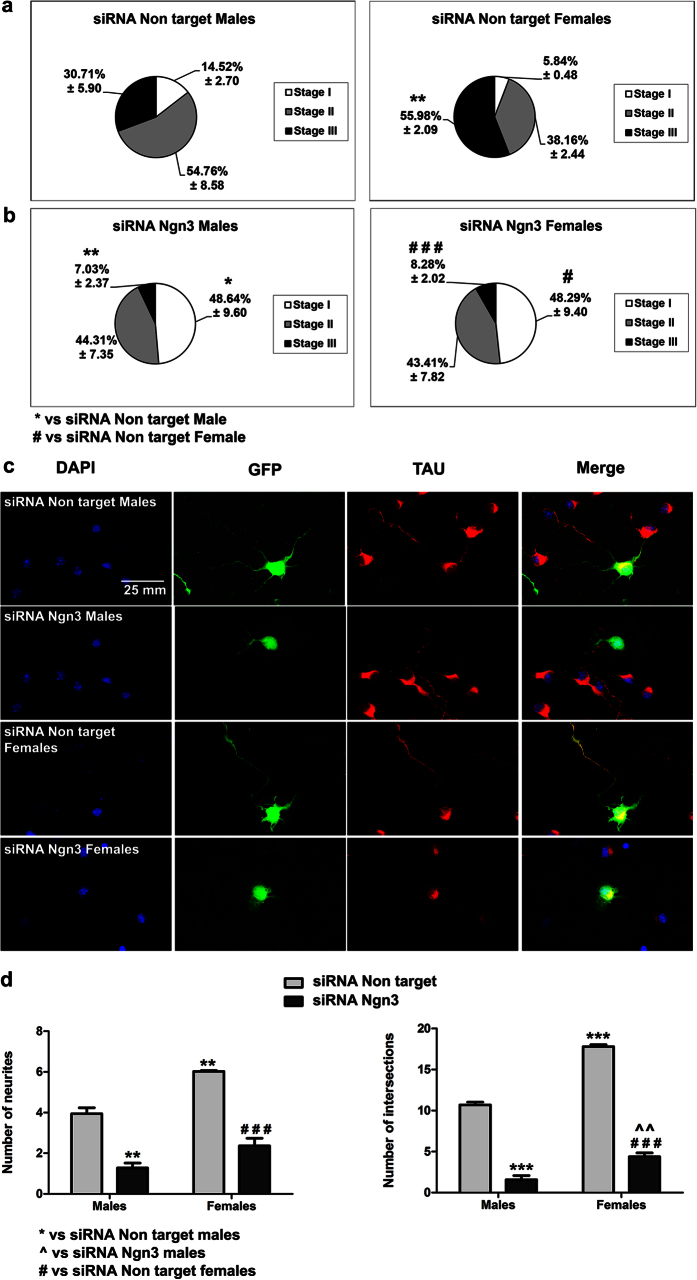
Neurogenin 3 (Ngn3) silencing decreases neuritogenesis in male and female neurons. (**a,b**) Proportion of neurons in the three stages of differentiation in male and female cultures at 2 DIV treated 24 h with non target or Ngn3 siRNA at 30 nM. (**c**) Representative examples of male and female neurons immunostained for Green Fluorescent Protein (GFP, green), TAU protein and DAPI (blue). (**d**) Number of primary dendrites and Sholl analysis. Data are the mean ± SEM of 3 hippocampal cultures ***p < 0.001 vs non target male values. ^###^p < 0.001 vs non target female values. ^^p < 0.01 vs Ngn3 SiRNA male values.

To determine whether Ngn3 was involved in neuritogenesis of male and female neurons, cultures were treated with a siRNA that was previously shown to decrease Ngn3 expression^13^. The efficacy of Ngn3 silencing was verified by Western blot showed in Supplementary Fig. S1. The band corresponding to the molecular weight of Ngn3 (23 kDa), decreases in the Ngn3 siRNA treatment respect to the siRNA non target in cultured hippocampal neurons. Treatment with the Ngn3 siRNA increased the proportion of neurons in Stage I in both male and female cultures compared to the cultures treated with the non target siRNA [Two-way ANOVA revealed an effect of treatment F(1,8) = 30.758; p = 0.001; Bonferroni: p = 0.049 in male and p = 0.015 in female] and decreased the proportion of neurons in Stage III [Two-way ANOVA showed an effect of sex F(1,8) = 14.369; p = 0.005, treatment F(1,8) = 104.107; p < 0.001 and a sex*treatment interaction [F(1,8) = 11.789; p = 0.009; Bonferroni: p = 0.008 in male and p < 0.001 in female]. In addition, Ngn3 silencing eliminated sex differences in the proportion of neurons in different stages of differentiation (Fig. 5a,b). In relation to the number of primary neurites, two-way ANOVA revealed a significant effect of sex [F(1,8) = 35.324; p < 0.001] and a significant effect of the treatment [F(1,8) = 140.300; p < 0.001] (Fig. 5c,d). In cultures treated with the non target siRNA, female neurons had a higher number of primary neurites than males (Bonferroni, p = 0.003). Treatment with the Ngn3 siRNA decreased the number of primary neurites in both male (p = 0.001) and female neurons (p < 0.001) vs non target siRNA values, abolishing the sex difference observed in the number of primary neurites (Fig. 5c,d). It was not possible to assess axonal length since neurons treated with the Ngn3 siRNA did not show Tau immunoreactive axons. For the Sholl analysis, Two-way ANOVA revealed a significant effect of sex [F(1,8) = 151.513; p < 0.001], treatment [F(1,8) = 783.152; p < 0.001] and a sex*treatment interaction [F(1,8) = 28.614; p = 0.001]. In the cultures treated with the non target siRNA, female neurons showed more intersections in the Sholl analysis than male neurons (Bonferroni, p < 0.001). The treatment with the Ngn3 siRNA decreased the number of neurite intersections in males (Bonferroni, p < 0.001) and females (p < 0.001) vs the non target siRNA values. However, sex differences in the number of intersections in the Sholl analysis were still observed after Ngn3 silencing (Bonferroni, p = 0.007). These findings suggest that Ngn3 is necessary for neuritogenesis in male and female neurons and further support that the enhanced expression of Ngn3 in female neurons may be the cause of their advanced development in comparison to male neurons.

### Aromatase inhibition reverses sex differences in neuritogenesis and Ngn3 expression

**Figure 6:**
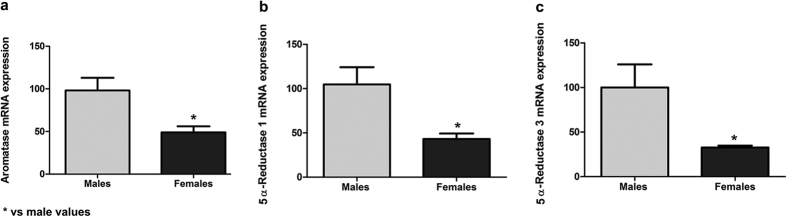
Aromatase, 5α—reductase 1 and 3 mRNA expression in male and female cultured hippocampal neurons. (**a**) Aromatase mRNA levels in male and female neurons at 1 DIV. Data are the mean ± SEM of 7 hippocampal cultures. (**b,c**) 5α—reductase 1and 3 mRNA levels in male and female neurons at 1 DIV. Data are the mean ± SEM of 5 hippocampal cultures. *p < 0.05 vs male values.

**Figure 7:**
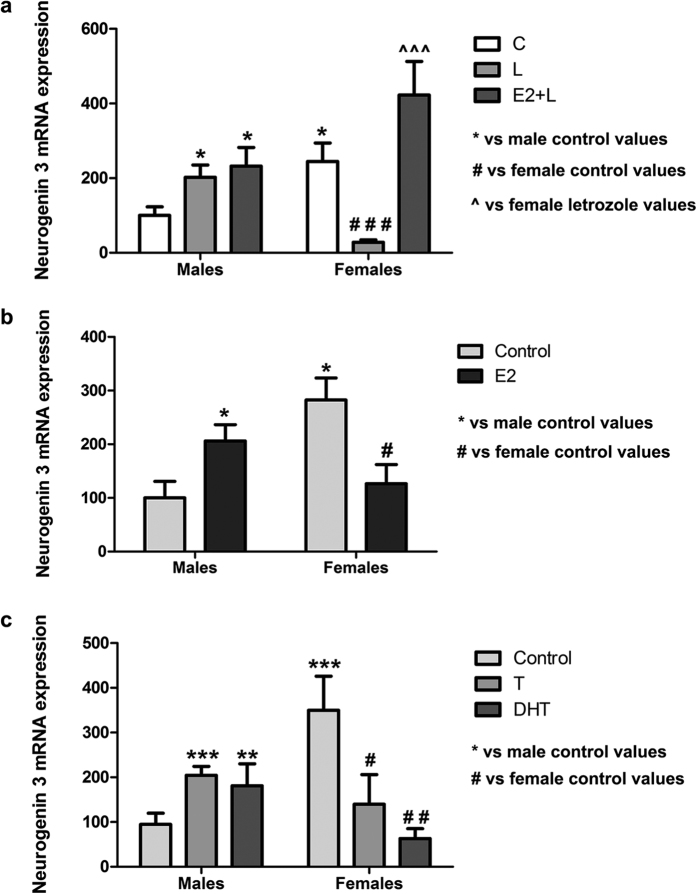
Aromatase inhibition, effect of oestradiol (E2), testosterone (T) and DHT in neurogenin3 (Ngn3) mRNA expression. (**a**) Ngn3 mRNA levels in male and female neurons at 1 DIV treated with letrozole (L) and L + E2. Data are the mean ± SEM of 5 hippocampal cultures. *p < 0.05 vs control male values. ^###^p < 0.001 vs control female values and ^^^p < 0.001 vs L female values. (**b**) Ngn3 mRNA levels in male and female neurons at 1 DIV treated with E2. Data are the mean ± SEM of 5 hippocampal cultures. *p < 0.05 vs control male values. ^#^p < 0.05 vs control female values. (**c)** Ngn3 mRNA levels in male and female neurons at 1 DIV treated with T and DHT. Data are the mean ± SEM of 6 hippocampal cultures. ***p < 0.001 and **p < 0.01 vs control male values. ^##^p < 0.01 and ^#^p < 0.05 vs control female values.

**Figure 8:**
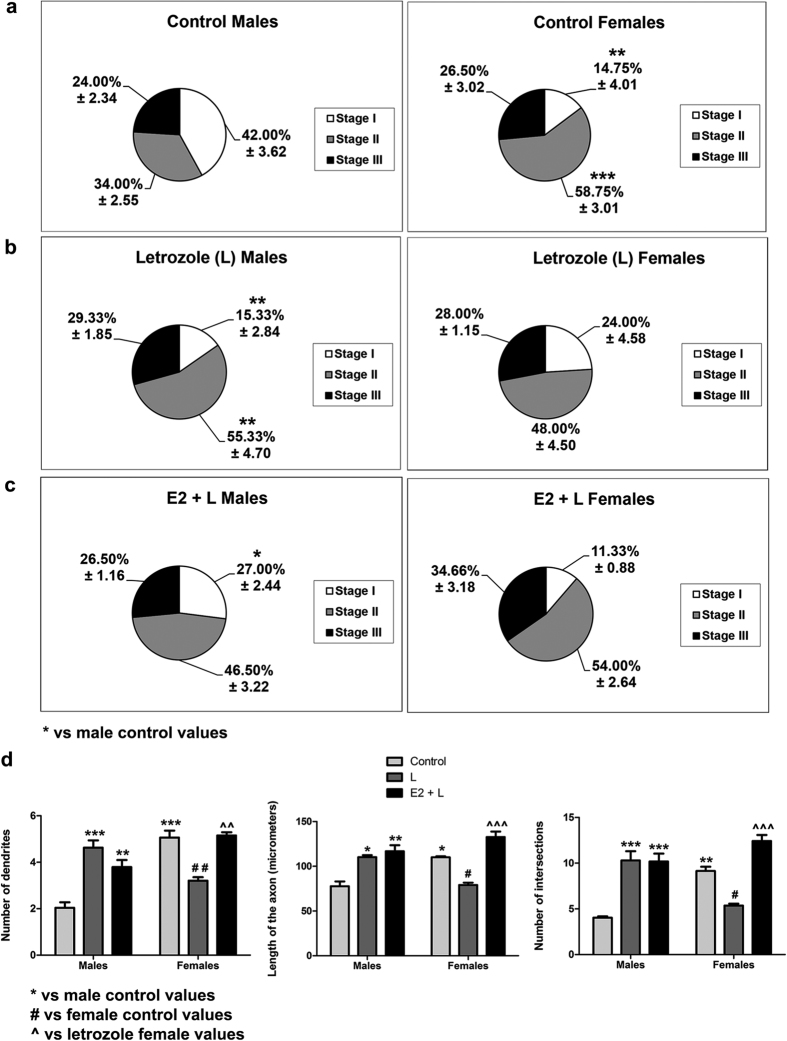
Aromatase inhibition reverses sex differences in neuritogenesis. (**a–c)** Proportion of neurons in the three stages of differentiation in male and female cultures at 2 DIV treated 24 h with E2 and L. (**d**) Number of primary neurites, length of the axon and complexity of the dendritic arbour (number of intersections of dendrites with the circles in the Sholl analysis). Data are the mean ± SEM of 3 hippocampal cultures. ***p < 0.001 **p < 0.01 and *p < 0.05 vs control male values. ^##^p < 0.01 and ^#^p < 0.05 vs control female values. ^^^p < 0.001 vs L female values.

Previous studies have shown that oestradiol increases Ngn3 expression and neuritogenesis in hippocampal neurons from unsexed embryos^13^,^14^. Since hippocampal neurons are capable of synthesizing oestradiol by the enzyme aromatase, we hypothesized that endogenous oestradiol may be involved in the generation of sex differences in Ngn3 and neuritogenesis. We first assessed aromatase expression in the cultures and confirmed that hippocampal neurons expressed aromatase. In addition, we observed that male cultures expressed higher aromatase mRNA levels than female cultures (Student’s t-test, p = 0.042; Fig. 6a). To determine whether aromatase activity was involved in the generation of sex differences in Ngn3 and neuritogenesis we treated the cultures with letrozole, an aromatase inhibitor (Figs 7 and 8). Two-way ANOVA revealed a significant effect of sex [F(1,49) = 10.011; p = 0.003], treatment [F(2,49) = 5.683; p < 0.006] and a significant sex*treatment interaction [F(2,49 = 7.794; p = 0.001] in Ngn3 mRNA levels. Letrozole increased Ngn3 expression (Bonferroni, p = 0.047) in male neurons but decreased Ngn3 expression in female neurons (p < 0.001) (Fig. 7a). Regarding the morphology in male neurons, letrozole treatment decreased the proportion of neurons in Stage I [Two-way ANOVA showed a significant effect of sex F(1,15) = 16.700; p = 0.001, treatment F(2,15) = 4.958; p = 0.022 and sex*treatment interaction F(2,15) = 13.982; p < 0.001; Bonferroni, p = 0.001] and increased the proportion of neurons in Stage II [Two-way ANOVA revealed and effect of sex F(1,15) = 8.746; p = 0.010 and a sex*treatment interaction F(2,15) = 11.050; p = 0.001; Bonferroni, p = 0.008] compared to control values (Fig. 8a,b). In contrast, in female neurons, letrozole did not affect the proportion of neurons in the different stages of differentiation (Fig. 8a,b). Concerning the morphology, two-way ANOVA revealed an effect of sex [F(1,13) = 21.051; p = 0.001], treatment [F(2,13) = 6.386; p = 0.012], and a sex*treatment interaction [F(2,13) = 34.723; p < 0.001] on the number of primary dendrites; an effect of treatment [F(2,13) = 25.221; p < 0.001] and a sex*treatment interaction [F(2,13 = 20.434; p < 0.001] in the length of axons; and an effect of treatment [F(2,13) = 26,220; p < 0.001] and sex*treatment interaction [F(2,13) = 27.282; p < 0.001], in the Sholl analysis (Fig. 8d). Letrozole increased the number of primary dendrites (Bonferroni, p < 0.001), the length of axons (p = 0.010) and the number of intersections (p < 0.001) in male neurons. In contrast, letrozole decreased the number of primary dendrites (p = 0.005), the length of axons (p = 0.015) and the number of intersections (p = 0.032) in female neurons (Fig. 8d). Representative examples of male and female neurons treated with letrozole are shown in Supplementary Fig. S2.

Aromatase inhibition by letrozole is expected to decrease oestradiol levels and to increase the levels of testosterone, the oestradiol precursor. We hypothesized that the different effect of letrozole on male and female neurons could be the result of a differential neuritogenic effect of testosterone and oestradiol. Thus, we decided to assess the effect of oestradiol and testosterone on the hippocampal cultures.

### Exogenous oestradiol promotes Ngn3 expression and neuritogenesis in male neurons but not in female neurons

**Figure 9:**
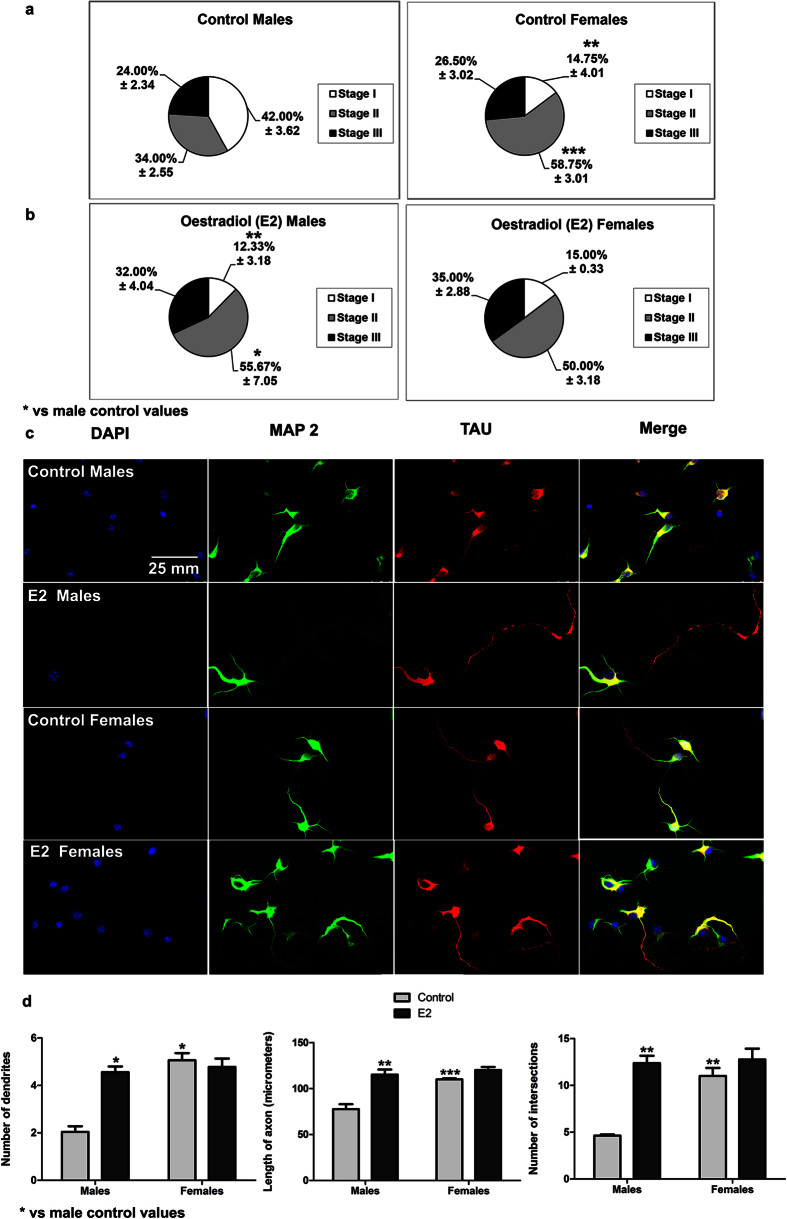
Effect of oestradiol (E2) on neuritogenesis of male and female hippocampal cultures. (**a,b**) Proportion of neurons in the three stages of differentiation in male and female cultures at 2 DIV treated 24 h with E2. (**c**) Representative examples of male and female neurons treated with E2 and immunostained for MAP 2 (green), TAU protein and DAPI (blue) at 2 DIV. (**d**) Number of primary neurites, length of the axon and complexity of the dendritic arbour (number of intersections of dendrites with the circles in the Sholl analysis). Data are the mean ± SEM of 3 hippocampal cultures. ***p < 0.001, **p < 0.01 and *p < 0.05 vs control male values.

Male and female hippocampal neurons were treated with oestradiol or vehicle. Two-way ANOVA revealed a sex*treatment interaction [F(1,14) = 14.487; p = 0.002] in Ngn3 mRNA levels (Fig. 7b); a treatment effect [F(1,10) = 8.477; p = 0.016] and sex*treatment interaction [F(1,10) = 11.637; p = 0.007] in the proportion of cells in Stage I; a sex effect [F(1,10) = 5.918; p = 0.035] and sex*treatment interaction [F(1,10) = 14.205; p = 0.004] in the proportion of cells in Stage II; an effect of sex [F(1,8) = 8.779; p = 0.018] in the number of primary dendrites; an effect of sex [F(1,8) = 33.955; p < 0.001], treatment [F(1,8) = 5.542; p = 0.046] and sex*treatment interaction [F(1,8) = 23.752; p = 0.01] in the length of the axons and an effect of sex [F(1,8) = 26.489; p = 0.001], treatment [F(1,8) = 7.468; p = 0.026] and sex*treatment interaction [F(1,8) = 22.261; p = 0.002] in the number of intersections (Fig. 9).

In hippocampal cultures originated from male embryos, the incubation with 10^−8^ M oestradiol resulted in an increase in Ngn3 mRNA levels (Bonferroni, p = 0.010) (Fig. 7b), in a reduction in the proportion of cells in Stage I (p = 0.007) and in a significant increase in the proportion of cells in Stage II (p = 0.020) (Fig. 9a,b). Oestradiol treatment also resulted in an increase in the number of primary dendrites (p = 0.025), the length of the axons (p = 0.006) and the number of intersections (p = 0.005) in male neurons (Fig. 9c,d). In contrast, in female neurons oestradiol induced a decrease in Ngn3 expression (p = 0.037) (Fig. 7b) without affecting the stages of neuronal differentiation and the neuritogenesis (Fig. 9). These findings indicate that exogenous oestradiol promotes neuronal differentiation in male but not in female neurons.

Previous studies have shown that oestradiol exogenously applied to hippocampal slice cultures had no effect on synapse density unless oestradiol synthesis was inhibited by using an aromatase inhibitor^15^. In view of this finding, we studied whether a similar mechanism underlies the failure of exogenous oestradiol to induce neuritogenesis in the cultures of female primary hippocampal neurons. We therefore treated male and female cultures with oestradiol in the presence of the aromatase inhibitor letrozole. In these cultures, application of oestradiol resulted in an increase in the expression of Ngn3 in female (Bonferroni, p < 0.001) neurons but not in male neurons compared to the cultures treated with letrozole alone (Fig. 7a). The treatment with oestradiol in the presence of letrozole had no effect in any stage of neuronal development in both sexes (Fig. 8b,c). Nevertheless, oestradiol treatment in the presence of letrozole increased the number of primary dendrites (Bonferroni p = 0.003), the axonal length (p < 0.001) and the number of intersections (p < 0.001) in female neurons compared to the cultures treated with letrozole alone but did not affect these parameters in male neurons (Fig. 7d). In female neurons treated with letrozole and oestradiol all the parameters examined reached levels similar to control values. This suggests that endogenous oestradiol is responsible of higher Ngn3 expression and neuritogenesis in female neurons than in males.

### Exogenous testosterone promotes Ngn3 expression and neuritogenesis in male neurons and decreases Ngn3 expression and neuritogenesis in female neurons

**Figure 10:**
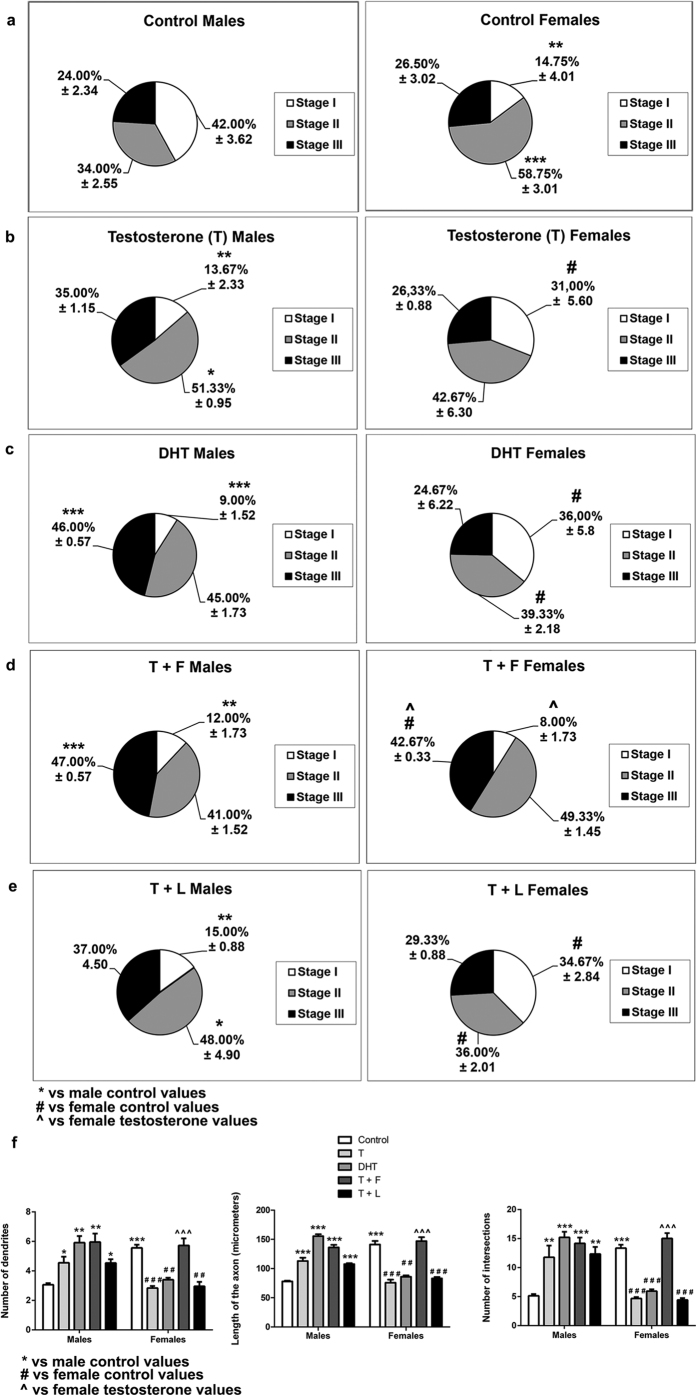
Effect of testosterone (T), dihydrotestosterone (DHT) finasteride (F) and letrozole (L) on neuritogenesis of male and female hippocampal neurons. (**a–e**) Proportion of neurons in the three stages of differentiation in male and female cultures at 2 DIV treated with T, DHT and T in presence of F or L. (**f**) Number of primary neurites, length of the axon and complexity of the dendritic arbour (number of intersections of dendrites with the circles in the Sholl analysis). Data are the mean ± SEM of 3 hippocampal cultures. ***p < 0.001, **p < 0.01 and *p < 0.05 vs control male values. ^###^p < 0.001 and ^##^p < 0.01 and ^#^p < 0.001 vs control female values. ^^^p < 0.001 vs T female values.

Male and female neurons were treated with 10^−7^M testosterone or vehicle. Two-way ANOVA revealed a sex*treatment interaction [F(2,30) = 26.435; p < 0.001] in Ngn3 mRNA levels (Fig. 7c); an effect of sex [F(1,22) = 4.808; p = 0.039], treatment [F(4,22) = 6.294; p = 0.002] and sex*treatment interaction [F(4,22) = 15.560] in the proportion of cells in Stage I; a sex*treatment interaction [F(4,22) = 13.298; p < 0.001] in the proportion of neurons in Stage II; an effect of sex [F(1,20) = 15.414; p = 0.001], treatment [F(4,20) = 14.872; p < 0.001], and sex*treatment interaction [F(4,20) = 21.848; p < 0.001] in the number of primary dendrites; an effect of sex [F(1,20) = 23.540; p < 0.001], treatment [F(4,20) = 41.240; p < 0.001], and sex*treatment interaction [F(4,20) = 74.238; p < 0.001] in the length of the axon and an effect of sex [F(1,20) = 25.726; p < 0.001], treatment [F(4,20) = 15.328; p < 0.001], and sex*treatment interaction [F(4,20) = 30.678; p < 0.001] in the number of intersections (Fig. 10).

Incubation of male neurons with 10^−7^M testosterone resulted in an increase in Ngn3 mRNA levels (Bonferroni, p < 0.01) (Fig. 7c), a reduction in the proportion of cells in Stage I (p = 0.002), an increase in the proportion of neurons in Stage II (Bonferroni, p = 0.032) (Fig. 10a,b), and an increase in the number of primary dendrites (p = 0.045), the length of the axon (p < 0.001) and the number of intersections (p = 0.004) (Fig. 10f). In contrast, in female neurons, application of testosterone resulted in a decrease in Ngn3 mRNA levels (p = 0.011; Fig. 7c), in a significant increase in the proportion of cells in Stage I (p = 0.046; Fig. 10a,b) and in a significant decrease in the number of primary dendrites (p < 0.001), the length of axons (p < 0.001) and the number of intersections (p < 0.001) vs control values (Fig. 10f).

The regulation of neuritogenesis by testosterone may be in part mediated by its metabolite oestradiol. However, in addition to be converted in oestradiol by the enzyme aromatase, testosterone can be converted in DHT by the enzyme 5α-reductase. Analysis of 5α-reductase expression in the cultures revealed that male and female hippocampal neurons expressed the 5α-reductase 1 and 3. However, we were unable to detect the expression of the isoform 2. In addition, we observed that male cultures expressed higher 5α-reductase 1 and 3 mRNA levels than female cultures (Student’s t-test, p = 0.041; Mann Whitney test, p = 0.015; Fig. 6b,c). In both male and female neurons, the active metabolite of testosterone DHT imitated the effect of testosterone on Ngn3 expression and neuritogenesis, increasing Ngn3 mRNA levels (Bonferroni, p = 0.006; Fig. 7c) and producing a reduction in the proportion of cells in Stage I (p < 0.001; Fig. 10a,c), and even an increase in the proportion of neurons in Stage III (p < 0.001; Fig. 10a,c) in male neurons while in females resulted in a significant decrease in Ngn3 mRNA expression (p = 0.006) (Fig. 7c) and in a significant increase in the proportion of cells in Stage I and a decrease in the proportion of cell in Stage II (p = 0.042 and 0.010, respectively; Fig. 10a,c). DHT also increased the number of primary dendrites (p = 0.005), the length of axons (p < 0.001) and the number of intersections (p < 0.001) in male neurons and decreased these parameters (p = 0.009; p < 0.001 and p = 0.001, respectively) in female neurons (Fig. 10f).

To determine whether the effect of testosterone on neuritogenesis was due to its conversion in DHT, we treated the cultures with finasteride, an inhibitor of 5α-reductase, the enzyme that converts testosterone in DHT. Finasteride blocked the effect of testosterone in female neurons but not in males. Treatment of female cultures with testosterone in the presence of finasteride decreases the proportion of the cells in Stage I (Bonferroni, p = 0.046), increases the proportion of cell in Stage III (p = 0.017) and increases the number of primary dendrites (p < 0.001), the length of the axon (p < 0.001) and the number of intersections (p < 0.001) vs testosterone treatment, reaching all these parameters similar levels to control values (Fig. 10b,d,f). Therefore, we may conclude that DHT mediates the effect of testosterone on the reduction of neuritogenesis in female neurons.

As mentioned before, testosterone can also be converted in oestradiol by the enzyme aromatase. Therefore, we analyzed the effect of letrozole, the inhibitor of the enzyme aromatase, on the effect of testosterone on neuritogenesis. Treatment of the cultures with letrozole did not modify the effects of testosterone, neither in male nor in female neurons (Fig. 10b,e,f). Examples of neurons treated with testosterone, DHT, testosterone + finasteride and testosterone + letrozole are shown in Supplementary Fig. S3. These findings suggest that testosterone promotes Ngn3 expression and neuritogenesis in male neurons by two different mechanisms, one that is mediated by its conversion in oestradiol and the other mediated by its conversion in DHT. This would explain that the inhibition of the conversion of testosterone in either DHT or oestradiol does not prevent the neuritogenic action of the hormone. The findings also suggest that the negative regulation of neuritogenesis by testosterone in female neurons is mediated by DHT.

### Steroid levels in male and female primary hippocampal cultures

The previous results indicate that oestradiol and DHT regulate Ngn3 and neuritogenesis in male and female neurons. To determine whether the levels of these steroids correlated with the sex difference in Ngn3 expression and neuritogenesis, steroids were assessed in the culture media of male and female neurons. As shown in Supplementary Table 1, the levels of testosterone, oestradiol, DHT and other steroids were not different between male and female cultures. This suggests that sex differences in Ngn3 expression and neuritogenesis are the consequence of sex differences in steroid signalling rather than the consequence of sex differences in steroid levels.

### Expression of steroid receptors in male and female neurons

**Figure 11:**
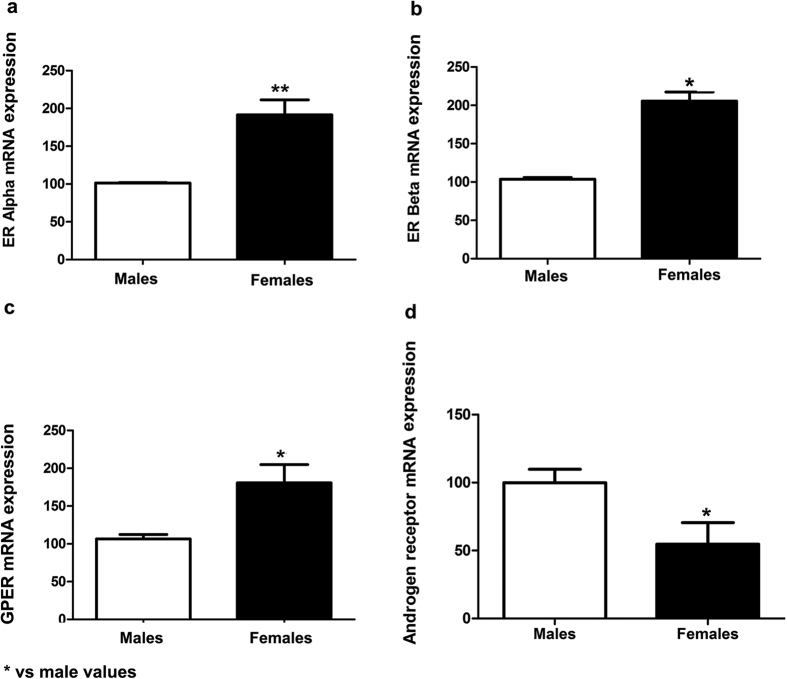
ER alpha (ERα), ER beta (ERβ), GPER and androgen receptor (AR) mRNA expression in male and female cultured hippocampal neurons. (**a**) ERα mRNA levels in male and female neurons at 1 DIV. (**b**) ERβ mRNA levels in male and female neurons at 1 DIV. (**c**) GPER mRNA levels in male and female neurons at 1 DIV. (**d**) AR mRNA levels in male and female neurons at 1 DIV. Data are the mean ± SEM of 4 hippocampal cultures for all the receptors measured. **p < 0.01 and *p < 0.05 vs control male values.

To determine whether male and female neurons differ in the expression of steroid receptors we assessed the mRNA levels of ERα, ERβ, GPER and androgen receptor (AR). As shown in Fig. 11, male and female neurons express ERα, ERβ, GPER and AR. The expression is higher in female neurons for the 3 oestrogen receptors (t-test p = 0.008, p = 0.014, p = 0.035, respectively); nevertheless, AR is more expressed in males than in females (p = 0.041).

## Discussion

Previous studies on unsexed primary neuronal cultures from the foetal mouse hippocampus have shown that there is a transitory peak in the expression of the neuritogenic factor Ngn3 that is associated with the earlier phases of neuritogenesis^12^,^16^. Our present findings indicate that the Ngn3 peak is advanced in female hippocampal neurons compared to males. In addition, we have observed that female neurons develop faster than male neurons. Thus, female hippocampal neurons at 2 DIV were at more advanced stages of differentiation than male hippocampal neurons under control conditions. In addition, we have identified Ngn3 as a factor involved in neuritogenesis of both male and female neurons and we have shown that the sex difference in neuronal development was associated with a higher expression of Ngn3 in female neurons than in male neurons during the first 2 DIV. Of note, Ngn3 expression was also higher in the hippocampus of females at E17 *in vivo*, suggesting that the sex-dependent differences of the neuritogenic process *in vitro* may reflect sex differences *in vivo*. These findings suggest that a temporal shift in the expression of Ngn3 is associated with a sex difference in neuronal development.

Previous studies in unsexed hippocampal primary neuronal cultures have shown that the treatment with oestradiol increases Ngn3 and neuritogenesis and that the silencing of Ngn3 blocked the effect of oestradiol^13^. Therefore, we hypothesized that oestradiol locally produced by female neurons may be the cause of their advanced expression of Ngn3 and their advanced development. Male and female neurons have steroidogenic activity and are able to synthesize the oestradiol precursor testosterone from cholesterol^3^,^17^. In addition, as shown here and in agreement with previous findings^18^,^19^,^20^, both male and female neurons express aromatase, the enzyme that converts testosterone in oestradiol. To test whether endogenous oestradiol is involved in the enhanced expression and development of female neurons at 2DIV, we incubated the cultures with letrozole, an inhibitor of aromatase. The inhibition of oestradiol synthesis with letrozole resulted in a decrease in Ngn3 expression and neuritogenesis in female neurons. Furthermore, the administration of exogenous oestradiol to the female neurons treated with letrozole recovered Ngn3 expression and neuritogenesis to control values. These findings support the hypothesis that endogenous oestradiol is involved in the enhanced expression of Ngn3 and neuritogenesis in female neurons.

The situation was very different in male neurons. The inhibition of oestradiol synthesis with letrozole in male cultures resulted in enhanced expression of Ngn3 and enhanced neuritogenesis. As female neurons, male neurons are also able to synthesize oestradiol and, indeed, they expressed higher levels of aromatase than female neurons, which is in agreement with previous findings in the literature showing that developing neurons from males possess a higher expression and activity of the enzyme than female neurons^21^. Therefore, the question was why letrozole did not decrease Ngn3 expression and neuritogenesis in male neurons? The inhibition of oestradiol synthesis with letrozole may cause not only a decrease in oestradiol levels but also an increase in the levels of the oestradiol precursor testosterone. Thus, we tested the hypothesis that testosterone caused an increase in Ngn3 and neuritogenesis in male neurons. Indeed, treatment of male neurons with testosterone resulted in an increase in Ngn3 and neuritogenesis. Surprisingly, however, testosterone decreased Ngn3 and neuritogenesis in female neurons. This was surprising because, as mentioned before, testosterone is a substrate for the enzyme aromatase and a precursor of oestradiol, which promotes Ngn3 expression and neuritogenesis in male neurons. Testosterone, however may also be converted to DHT by the enzyme 5α-reductase, which is expressed in the brain, including the hippocampus^22^ and as shown here in primary hippocampal neurons. We measured the 3 isoforms of the enzyme 5α-reductase and we observed that the isoforms 1 and 3 were expressed in the hippocampal cultures. As observed for aromatase levels, the mRNA levels of both 5α-reductase isoforms were higher in male neurons, probably because male neurons are primed to metabolize testosterone produced by foetal testis to oestradiol and DHT. Since both male and female hippocampal neurons expressed aromatase and 5α-reductase, we decided to study the effect of the two metabolites of testosterone, oestradiol and DHT, in both sexes.

Oestradiol increased Ngn3 expression and neuritogenesis in male neurons. However, oestradiol did not affect Ngn3 expression and neuritogenesis in female neurons. There are several possible explanations for the different response of male and female neurons to exogenous oestradiol in the regulation of neuritogenesis. An important consideration is that female neurons were more differentiated and expressed higher levels of Ngn3 than male neurons when exposed to oestradiol, being therefore potentially less responsive to this hormone. In addition, our findings, showed that the aromatase inhibitor letrozole decreased Ngn3 expression and neuritogenesis in female neurons and that exogenous oestradiol was able to promote Ngn3 expression and neuritogenesis when these cells were treated with letrozole. This suggests that the intrinsic production of oestradiol by female neurons causes both their insensitivity to exogenous oestradiol and their higher morphological differentiation. In other words, a ceiling effect of endogenous oestradiol may account for the ineffectiveness of exogenously applied oestradiol to further promote neuritogenesis. A similar situation has been observed in female rat hippocampal organotypic cultures, where local production of oestradiol prevents the action of exogenous oestradiol on the formation of dendritic spine synapses^15^,^23^. Since male neurons expressed lower levels of Ngn3 than female neurons, oestradiol treatment was effective in increasing Ngn3 expression and neuritogenesis. These findings suggest that oestradiol promotes Ngn3 and neuritogenesis in both male and female neurons, but endogenous oestradiol is more active in females, at least in the first day in culture.

Oestradiol activates neuritogenesis by a variety of signalling mechanisms that may involve oestrogen receptor α (ERα), oestrogen receptor β (ERβ) or G protein-coupled oestrogen receptor 1 (GPER)^1^. Differences in oestradiol signalling in male and female neurons may be the cause of the observed sex difference in the neuritogenic activity of endogenous oestradiol, since oestradiol levels under basal conditions were not different between male and female hippocampal primary neuronal cultures. In contrast, the expression of ERs under basal conditions was higher in female neurons than in male neurons.

**Figure 12:**
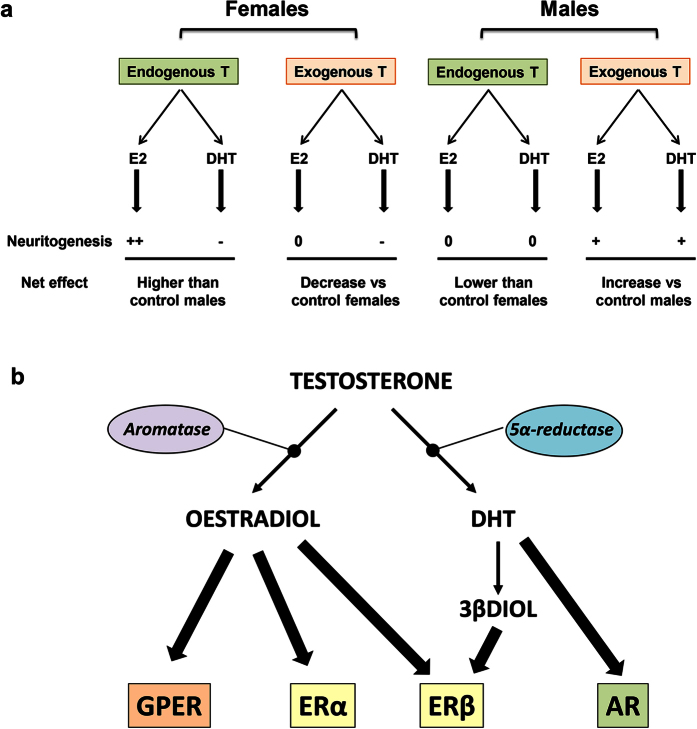
Summary of the effect of testosterone on neuritogenesis in male and female hippocampal neurons. (**a**) Exogenous and endogenous effect of testosterone (T) and their metabolites oestradiol (E2) and dihydrotestosterone (DHT) on neuritogenesis in male and female hippocampal neurons. T is transformed in E2 and DHT in male and female neurons. In females, E2 produced from endogenous T (control conditions) increases neuritogenesis, while DHT produced from endogenous T decreases neuritogenesis. The net result is an increased neuritogenesis compared to control male neurons. Also in females, E2 produced from exogenous T (hormonal treatment) is unable to further increase neuritogenesis over the values induced by E2 produced from endogenous T. In contrast, DHT produced by exogenous T is able to reduce the neuritogenic action of E2 produced from endogenous T. The net result is a decreased neuritogenesis compared to control female neurons. In males, E2 and DHT produced by endogenous T are unable to promote neuritogenesis to the levels of control female neurons. In contrast, E2 and DHT produced from exogenous T promote neuritogenesis in male neurons over control male values. (**b**) Proposed model of the receptors involved in the actions of testosterone in male and female hippocampal neurons. In both male and female neurons testosterone is transformed in oestradiol and DHT. Oestradiol acts on oestrogen receptors (GPER, ERα and ERβ), while DHT acts on androgen receptor (AR). In addition, DHT may be converted in 3βDIOL, an agonist of ERβ. The different transcriptional activity of each oestrogen receptor form, together with the different expression of oestrogen and androgen receptors in male and female neurons may be involved in the sex dimorphic neuritogenic actions of endogenous and exogenous testosterone.

The other metabolite of testosterone, DHT, also had different effects on neuritogenesis in male and female neurons. DHT increased Ngn3 expression and neuritogenesis in male neurons but decreased Ngn3 expression and neuritogenesis in female neurons. Furthermore, in cultures treated with the inhibitor of DHT synthesis finasteride, testosterone was not able to decrease Ngn3 expression and neuritogenesis in female neurons, further suggesting that the effect of testosterone in decreasing neuritogenesis in female neurons was mediated by DHT. In contrast, the inhibition of DHT synthesis in male neurons did not prevent the neuritogenic action of testosterone, probably because testosterone was also exerting its neuritogenic action in male neurons through its conversion to oestradiol. Figure 12a,b shows a summary of the interpretation of the results.

As mentioned before, the different expression of ERs in male and female neurons may contribute to the sex dimorphic effects of endogenous oestradiol. Furthermore, it has been found that oestradiol may regulate the same function in the male and female brain using a different set of ERs in each sex. For instance, oestradiol induces synaptic potentiation in the hippocampus promoting glutamate release via ERβ in female synapses and via ERα in male synapses. In addition, in the postsynaptic side, oestradiol increases glutamate sensitivity via GPER in female synapses and via ERβ in male synapses^24^. Whether oestradiol is activating a different set of ERs in male and female neurons to promote Ngn3 expression and neuritogenesis merits future investigation. On the other hand, ER signalling interacts with the signalling of the receptors for other factors, such as insulin like growth factor-I, Wnt or Notch^25^, whose expression and activity may differ in male and female neurons^26^,^27^,^28^.

In addition, our findings suggest that DHT also activates different mechanisms in male and female neurons to regulate Ngn3 expression and neuritogenesis. DHT acts on androgen receptors, whose expression is higher in male than in female cultured hippocampal neurons, and is also metabolized in molecules with affinity for ERβ, such as 5α-androstane-3β,17β-diol (3β-DIOL)^29^,^30^,^31^ 3β-DIOL may have a different effect on Ngn3 expression and neuritogenesis than oestradiol. It is important to consider that oestradiol binds to ERα, ERβ and GPER and that the transcriptional regulation exerted by these receptors may have different and even opposite effects in the expression of some genes^29^,^32^,^33^,^34^,^35^. For instance, oestradiol and testosterone have been shown to regulate arginine-vasopressin expression in female neuroblastoma cells exerting a stimulatory action via ERα and an inhibitory action via ERβ^29^. Further studies are necessary to determine the signalling mechanisms involved in the different action of DHT in male and female neurons as well as the role of each ER subtype in the regulation of Ngn3 expression and neuritogenesis (see Fig. 12b for a summary of the possible steroid receptors involved in the regulation of neuritogenesis). It should be noted also that we did not explore the mechanism involved in the peak of Ngn3 at 2 DIV in male neurons, which may also depend on the local actions of oestradiol and DHT.

The most relevant finding in our study is that oestradiol produced by female neurons, in absence of exogenous hormonal steroids, participates in the generation of sex differences in neuronal development. However, one of the major limitations of our study is that it is mainly based on experiments in cultured neurons. Further studies are necessary to determine the role exerted by oestradiol synthesized by female neurons on hippocampal development *in vivo*. Another question that remains to be determined is the long-term functional significance of the observed sex difference in neuronal development, since sex differences in Ngn3 expression *in vivo* and *in vitro* were transient. Although sex differences in Ngn3 expression are not persistent during development, a different timing in the maturation of male and female neurons may result in a different pattern of synaptic connectivity, due to a different matching between the maturation of presynaptic and postsynaptic structures. It is important to emphasize that the synaptic network of hippocampal interneurons is under development at late embryonic days in rodents, when the transient sex difference in Ngn3 levels occurs in the hippocampus *in vivo*^36^,^37^,^38^,^39^. Thus, the existence of transient sex differences in neuronal development during this critical period may result in the generation of a different functional synaptic connectivity in the hippocampal circuits that may participate in the reported sex differences in hippocampal function during adult life^40^,^41^,^42^,^43^,^44^. Further studies are necessary to validate this hypothesis.

The best characterized cause for the generation of sex differences in brain structure and function in rodents is the organizational action of testosterone during critical periods of development^45^. The action of testosterone is in part mediated by its intracerebral conversion in oestradiol or DHT in the male brain^46^. The participation of female neuron-derived oestradiol in the generation of sex differences in neuritogenesis challenges the classical view according to which brain sex differences in mammals are the result of the organizational action of testosterone in males. More recent studies suggest the existence of multiple parallel mechanisms for mammalian brain sex differentiation, including the direct action of factors encoded by sex chromosomes^47^ and the active repression of masculinisation in the female brain^48^. Our present findings indicate that female neurons play an active role in the generation of sex differences in neural tissue via the local synthesis of oestradiol.

## Methods

### Animals

CD1 mice were raised at the Cajal Institute and used to generate embryos for this study. The day of vaginal plug was defined as embryonic day 0. All procedures for handling and killing the animals used in this study were in accordance with the European Commission guidelines (86/609/CEE and 2010/63/UE) and the Spanish regulation (R. D. 53/2013) on the protection of animals for experimental use and were approved by our institutional animal care and use committee (Comité de Experimentación Animal del Instituto Cajal) and the Consejeria del Medio Ambiente y Territorio (Comunidad de Madrid, Ref. PROEX 200/14).

### Genotyping

Genotyping was performed on genomic DNA samples of E17 brain cortex from mouse embryos by PCR for the Sry transgene. PCR reaction were prepared by using Supreme NZTY-taq Master Mix(2x) and the following primers: SryF (forward): 5′-TTGTCTAGAGAGCATGGAGGGCCATGT CAA-3′; SryR (reverse): 5′-CCACTCCTCTGTGACACTTTAGCCCTCCGA-3′^49^. PCR was then carried out using a thermocycler and the PCR products were separated by gel electrophoresis and visualized.

### Hippocampal Neuronal Cultures and Experimental Conditions

The hippocampi were dissected out from embryonic day 17 male and female mouse embryos and dissociated to single cells after digestion with trypsin (Worthington Biochemicals, Freehold, NJ) and DNase I (Sigma-Aldrich)^50^. Neurons were plated on 6-wells plates or glass coverslips coated with poly-L-lysine (Sigma-Aldrich) at a density of 20 neurons/mm^2^ for morphological studies and a density of 700 neurons/mm^2^ for mRNA, protein and steroid measurements. Cells were cultured in phenol red free Neurobasal medium supplemented with B-27, GlutaMAX I and antibiotic/antimycotic (Invitrogen, Crewe, United Kingdom). Under these conditions, we assessed glial growth at 1 or 2 DIV. The level of astrocyte contamination was less than 5%, judged by immunocytochemistry for glial fibrillary acidic protein, neuron-specific βIII tubulin and 4′,6-diamidine-2′-phenylindole dihydrochloride (DAPI) nuclear staining.

### Cell treatments

After 1 DIV the culture medium was replaced for 2 h by fresh medium devoid of B27 and GlutaMAX I supplement for Western blotting and quantitative real-time polymerase chain reaction analysis. We treated the cells for 2 h with the following test compounds, alone or in combination in the same medium: testosterone (10^−7^ M; Sigma-Aldrich); 17β-oestradiol (10^−8^ M; Sigma-Aldrich), the aromatase inhibitor letrozole (10^−8^ M; Tocris BioScience, Bristol, UK) or the 5α-reductase inhibitor finasteride (10^−7^ M; Sigma-Aldrich). The doses of steroids and inhibitors are based in previous studies^13^,^24^. The test compounds were dissolved in dimethyl sulfoxide and then to the final concentration in Neurobasal medium. For immunocytochemistry assays, the treatments were performed over a period of 24 h, and cultures were not deprived of B27 and GlutaMAX I supplement.

### Transfection

Neurons were transfected at 1 DIV using the Effectene Transfection Reagent (Qiagen GmbH, Hilden, Germany), following the manufacturer’s instructions. Cells were co-transfected with pEGFP-C2 plus small interfering RNA (siRNA) oligonucleotide targeted to Ngn3. After 20 h of expression time the cultures were fixed in 4% paraformaldehyde in 0.1 M phosphate buffer for immunostaining.

The siRNA oligonucleotides were purchased from Applied Biosystems/Ambion and the concentration was 30 nM during transfection. SiRNAs targeting Ngn3 was: Ngn3-siRNA (sense, AACUACAUCUGGGCACUGAtt; antisense, UCAGUGCCCAGAUGUAGUUgt). A non-targeting siRNA was used as negative control (Silencer^®^ Select Negative Control #1 siRNA, catalogue number 4390843).

### Immunocytochemistry

Cells at 2 DIV were fixed for 20 min at room temperature in 4% paraformaldehyde and permeabilized for 4 min with 0.12% Triton-X plus 0.12% gelatin in phosphate buffered saline (PBS). Cells were then washed with PBS/gelatin and incubated for 1 h with chicken anti-microtubule associated protein-2 (MAP2) (Abcam, Cat# ab75713, diluted 1:10.000 in PBS/gelatin) and rabbit anti-TAU (Abcam, Cat# ab47568, diluted 1:500 in PBS/gelatin) or goat anti-GFP (Abcam, Cat# ab5450, diluted 1:1000 in PBS/gelatine) to enhance the fluorescence of the transfected cells. After washing in the same buffer, cells were incubated for 1 h at room temperature with Alexa fluor donkey anti chicken (Jackson Immunoresearch, Cat# 703-545-155) and Alexa 594-donkey anti- rabbit (Jackson Immunoresearch, Cat# 711-585-152 diluted 1:1000 in PBS/gelatin). Cell nuclei were stained with DAPI.

### Morphometric analysis

To assess the effect of Ngn3 siRNA, oestradiol, testosterone, DHT, finasteride and letrozole on cultured hippocampal neuronal morphology, neurites or dendrites were stained at 2 DIV with anti-GFP or anti-MAP2 antibodies and axons were labelled with anti-TAU 1 antibody. We classified the neurons in 3 stages of differentiation based on their morphology. Stage I corresponds to a neuron without neurites and axon; Stage II corresponds to a neuron with primary neurites or defined dendrites but without axon; Stage III corresponds to a neuron with a high or low number of dendrites with a defined axon. Dendrites were counted in neurons in Stages II and III. The proportion of neurons in each stage was determined by assessing 100 neurons per experimental condition. In addition, the neuritic arbor was assessed by Sholl analysis^51^ using a grid of 6 concentric circles with increasing radius of 5 μm. The grid was superimposed on the neurons stained with anti-GFP, anti-MAP2 and anti-TAU 1 at a magnification of 400X, placing the innermost circle over the perikaryon. For the siRNA experiments, the number of neurites intersecting each circle was counted in 20–30 cells per experimental condition. In the treatments with oestradiol, testosterone, DHT, finasteride and letrozole, the number of dendrites intersecting each circle was measured only in neurons in Stages II and III. The length of axons was measured in neurons at Stage III using the Image J program (freely available at: imagej.nih.gov).

### Analysis of gene expression by quantitative real-time polymerase chain reaction (q-PCR)

Total RNA was extracted from cultures with illustra RNAspin Mini RNA isolation kit from GE Healthcare (Buckinghamshire, UK). First strand cDNA was prepared from RNA using the Moloney murine leukemia virus reverse transcriptase (Promega Corp., Madison, Wisconsin) following the manufacturer’s instructions. Quantitative PCR reactions were carried out on an ABI Prism 7000 Sequence Detector (Applied Biosystems, Weiterstadt, Germany) using the TaqMan or Sybr Green Universal PCR Master Mix. TaqMan probes and primers for Ngn3 and 5α-Reductase 1, 2 and 3 were Assay-on-Demand gene expression products (Applied Biosystems). Primer sequences for aromatase, ERα, ERβ, GPER, androgen receptor (AR) and the control housekeeping gene 18 S rRNA, were designed using Primer Express (AB) (Table 1).

### Western blotting

Proteins were resolved by SDS-PAGE and transferred onto nitrocellulose Trans-Blot Turbo membranes from Applied Biosystems (Weiterstadt, Germany). The membranes were blocked in Tris-buffered saline containing 0.1% Tween 20 and 5% bovine foetal serum and incubated first with primary antibodies and then with horseradish peroxidase-conjugated secondary antibodies. The following primary antibodies were used: anti-Ngn3 rabbit polyclonal antibody (Abcam, Cat# ab38548, diluted 1:500) and anti-glyceraldehyde 3-phosphate dehydrogenase mouse monoclonal antibody (dilution 1:1000, Millipore, Cat# MAB374). All secondary antibodies were from Jackson Immuno Research (West Grove, PA, USA). Specific proteins were visualized with enhanced chemiluminescence detection reagent according to the manufacturer’s instructions (Amersham, GE Healthcare Europe, Barcelona, Spain). The quantification of protein was carried out by densitometric analysis using the Image J program (freely available at: imagej.nih.gov).

### Quantitative analysis of steroids by liquid chromatography and mass spectrometry

Male and female neurons were plated on 6-wells plates at a density of 700 neurons/mm^2^ and cultured in phenol red free Neurobasal medium supplemented with B-27 and GlutaMAX I. Cells were incubated in this medium for 24 h. and then the medium was collected and stored at −80 °C until analyzed. Extraction and purification of the samples were performed according to Caruso and colleagues^52^ with modifications. Briefly, samples were spiked with ^13^C_3_-17β-Estradiol (2 ng/sample), ^13^C-Progesterone (0.4 ng/sample) and ^13^C-Pregnenolone (10 ng/sample), as internal standards. After an overnight extraction at 4 °C in the presence of 3 volumes of 1% acetic acid in methanol, samples were centrifuged at 12,000 rpm for 5 min and the pellet was extracted twice with 1 ml of MeOH/acetic acid (99:1 v/v). The organic phases were combined and passed through SPE cartridges, as previously described^52^. Briefly, blank samples were spiked with internal standards and increasing amounts (0.05–5 ng/sample) of each steroid were added. Calibration curves were extracted and analyzed as described for experimental samples.

Positive atmospheric pressure chemical ionization experiments were performed with a linear ion trap - mass spectrometer (ThermoElectron Co, San Jose, CA, USA) using nitrogen as sheath, auxiliary and sweep gas. The instrument was equipped with a Surveyor liquid chromatography Pump Plus and a Surveyor Autosampler Plus (ThermoElectron Co, San Jose, CA, USA). The mass spectrometer was employed in tandem mode using helium as collision gas. Mobile phases were previously described^52^. The Hypersil Gold column (100 × 3 mm, 3 μm; ThermoElectron Co, San Jose, CA, USA) was maintained at 40 °C. Peaks were evaluated using a Dell workstation by means of the software Excalibur^®^ release 2.0 SR2 (ThermoElectron Co, San Jose, CA, USA). Samples were analyzed using the transitions previously reported^52^.

### Statistical analysis

Statistical significance was assessed by two-way analysis of variance (ANOVA) with sex and treatment as factors followed by the Bonferroni post-hoc test. When there were only two experimental groups, data was analyzed using the Student’s t-test or Mann–Whitney U-test. The SPSS 19.0 software package (SPSS, Inc., Chicago, IL, USA) was used for statistical analysis. The levels of significance were denoted as *^, #, $^p < 0.05, **^, ##,^ ^^p < 0.01, ***^, ###,^ ^^^p < 0.001. The number used for statistical analysis was the number of independent experiments (independent cultures obtained from embryos of different mothers) and it is indicated in the figure legends.

## Additional Information

**How to cite this article**: Ruiz-Palmero, I. *et al*. Oestradiol synthesized by female neurons generates sex differences in neuritogenesis. *Sci. Rep.* **6**, 31891; doi: 10.1038/srep31891 (2016).

## Supplementary Material

Supplementary Information

## Figures and Tables

**Table 1:** Primer sequences for real-time polymerase chain reaction.

Primer	Forward 5′-3′	Reverse 5′-3′
Aromatase	GGATGTTGTTGACCCTCATGAGAC	GATGTTTGGTTTGATGAGGAGAGC
ERα	GATCCCACCATGCACAGTGA	GGAGCATCTACAGGAACACAGGTA
ERβ	CCTGGTCTGGGTGATTTCGA	ACTGATGTGCCTGACATGAGAAAG
GPER	TGCTGCCATCCAGATTCAAG	GGGAACGTAGGCTATGGAAAGAA
AR	TCTACTTTGCACCTGACTTGGTTT	ACTCTTGAGACAGGTGCCTCATC
18 S rRNA	CGCCGCTAGAGGTGAAATTCT	CATTCTTGGCAAATGCTTTCG

Real-time PCRs were performed following the supplier’s instructions using the TaqMan or Sybr Green Universal PCR Master Mix. All reactions were done in duplicates, from at least 4 different cultures. Ngn3 expression was normalized for 18 S rRNA expression. The ΔΔCT method was used for relative quantification analysis.
