# Supplementary material for: Oestradiol synthesized by female neurons generates sex differences in neuritogenesis
Source: Sci Rep. 2016 Aug 24;6:31891. doi: 10.1038/srep31891 (PMC4995407; doi:10.1038/srep31891)
Supplement: Supplementary Information [file srep31891-s1.pdf]

Supplementary Information for:

Oestradiol synthesized by female neurons generates sex differences in neuritogenesis

Isabel Ruiz-Palmero[1], Ana Ortiz-Rodriguez[1], Roberto Cosimo Melcangi[2], Donatella Caruso[2], Luis M. Garcia-Segura[1,*], Gabriele M. Rune[3] and Maria-Angeles Arevalo[1]

[1] Instituto Cajal, Consejo Superior de Investigaciones Científicas (CSIC), Avenida Doctor Arce 37; 28002 Madrid, Spain

[2] Dipartimento di Scienze Farmacologiche e Biomolecolari, Center of Excellence on Neurodegenerative Diseases, Università degli Studi di Milano,Via G. Balzaretti 9, 20133 Milan, Italy

[3] Institute of Neuroanatomy, University Medical Center Hamburg-Eppendorf, Martinistr. 52, 20246 Hamburg, Germany

*** Corresponding author:** lmgs@cajal.csic.es

Supplementary Fig. S1

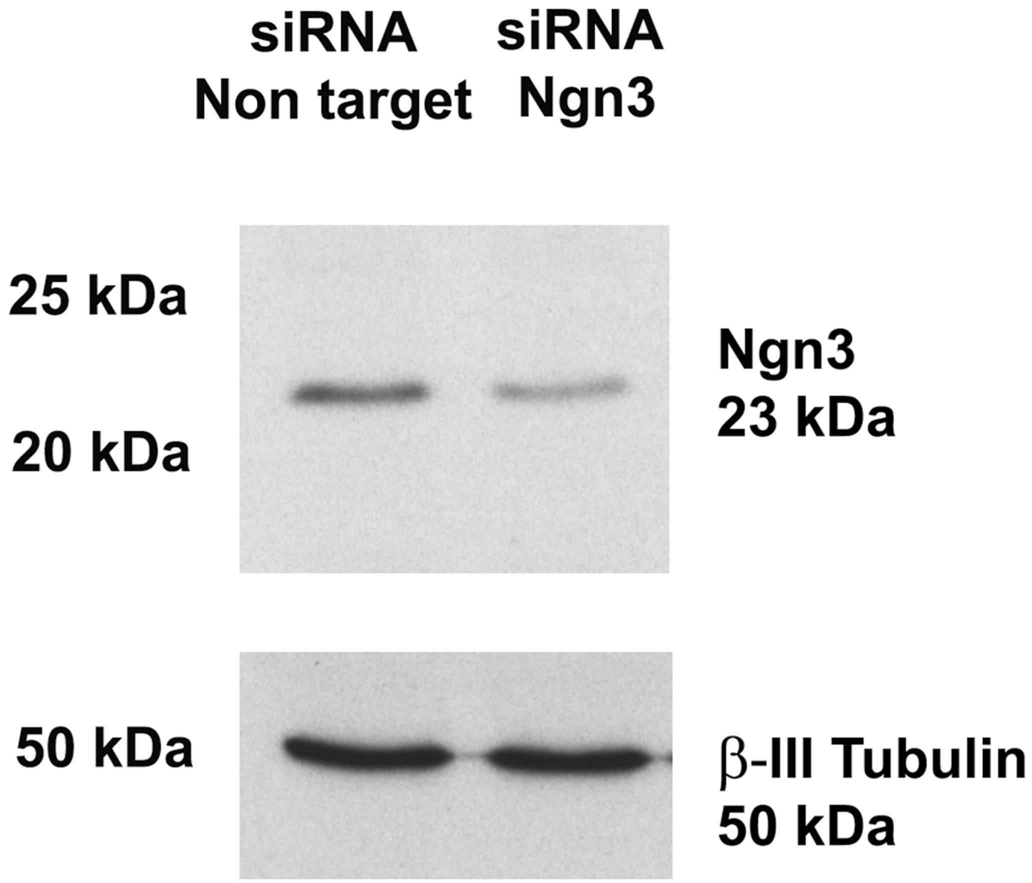

Supplementary Fig. S1. Downregulation of Ngn3 expression by the action of a specific Ngn3 siRNA. The efficacy of Ngn3 silencing was verified by Western blot. The band corresponding to the molecular weight of Ngn3 (23 kDa), decreases after the Ngn3 siRNA treatment respect to the non target siRNA in cultured hippocampal neurons.

Supplementary Fig. S2

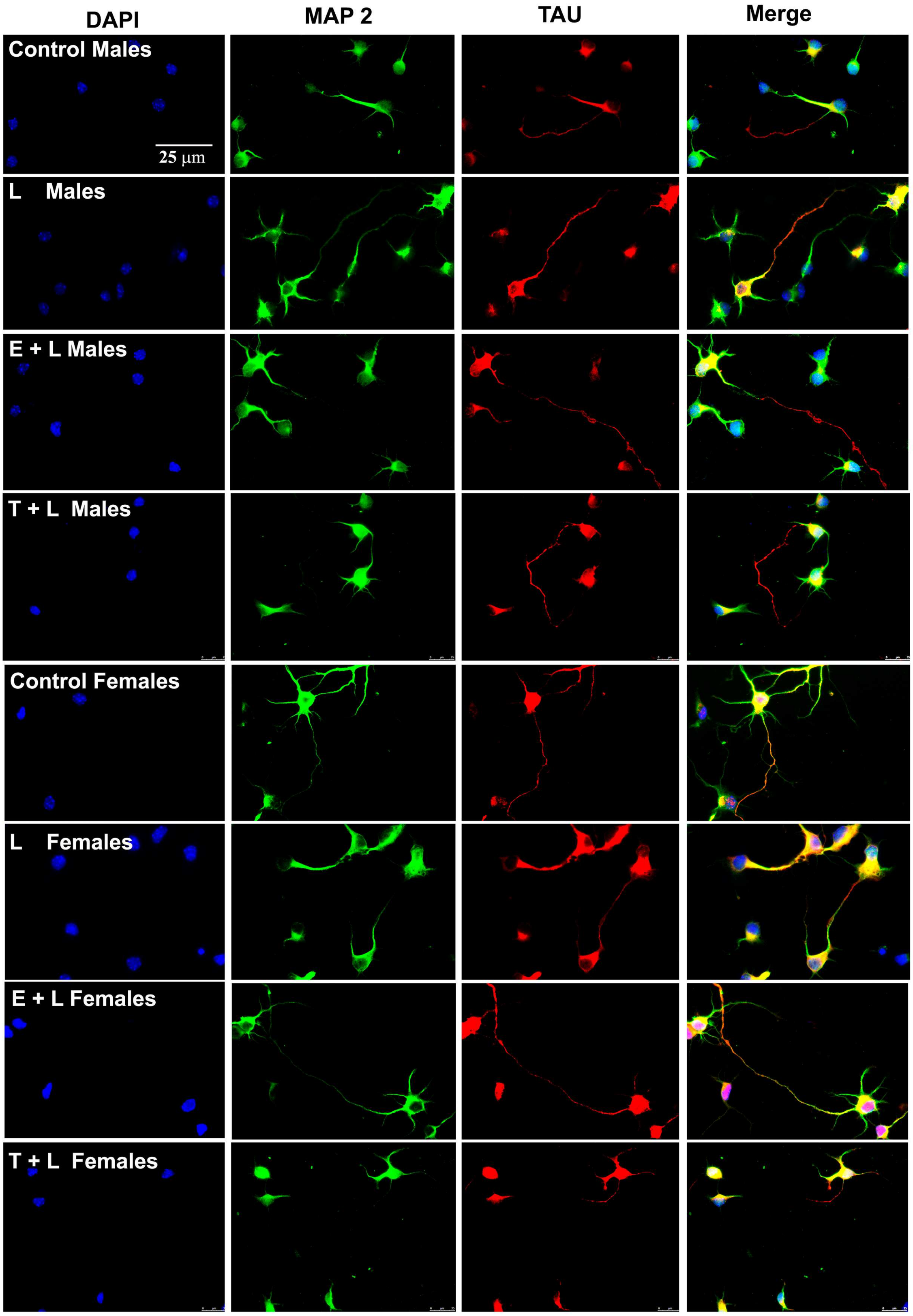

Supplementary Fig. S2. Representative examples of male and female neurons treated with letrozole (L). Hippocampal neuronal cultured were treated at 1 DIV with L, L+E2 and L+T and immunostained for MAP 2 (green), TAU (red) and DAPI (blue) at 2 DIV.

Supplementary Fig. S3

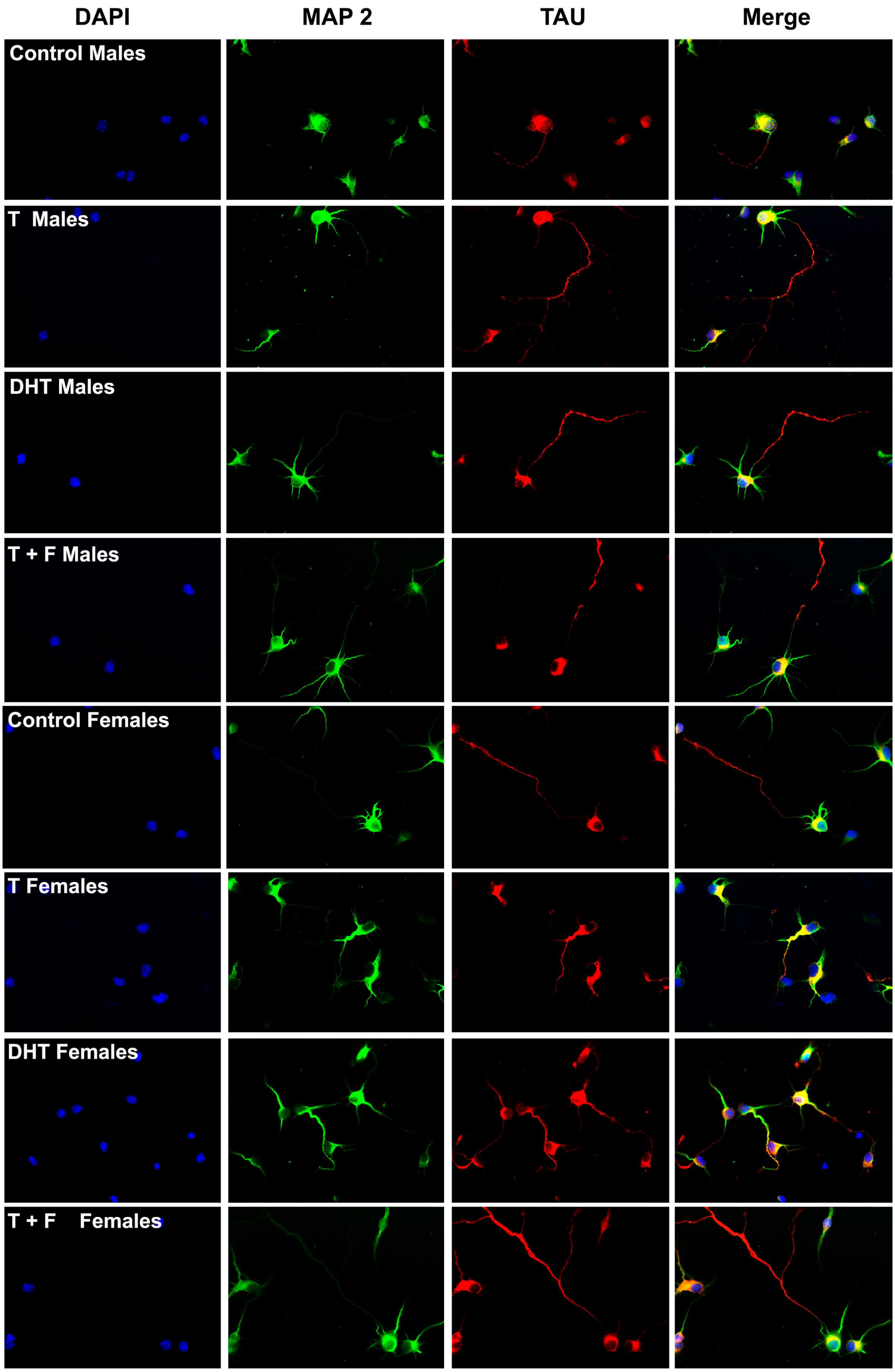

Supplementary Fig. S3. Representative examples of male and female neurons treated with testosterone (T), dihydrotestosterone (DHT) and finasteride (F). Hippocampal neuronal cultured were treated at 1 DIV with T, DHT and T+F and immunostained for MAP 2 (green), TAU (red) and DAPI (blue) at 2 DIV.

Supplementary Table 1. Levels of steroids in the culture medium of male and female hippocampal neurons from E17 embryos

Steroid	Sex	Levels in culture medium (pg/ml)
Pregnenolone	Males<br>Females	9.136 ± 1.764<br>12.02 ± 1.852
Progesterone	Males<br>Females	2404 ± 216<br>2393 ± 200
Dihydroprogesterone	Males<br>Females	2026 ± 98<br>2034 ± 85
Isopregnanolone	Males<br>Females	61.92 ± 12.79<br>63.81 ± 8.319
Allopregnanolone	Males<br>Females	39.65 ± 8.371<br>32.09 ± 1.854
Testosterone	Males<br>Females	2.349 ± 0.420<br>2.720 ± 0.403
Dihydrotestosterone	Males<br>Females	2.840 ± 1.226<br>1.280 ± 0.631
Oestradiol	Males<br>Females	4.816 ± 0.594<br>4.253 ± 0.843

Data are mean±SEM from 8 cultures.
